# Aetiology and Clinical Presentation of Windswept Deformity in Ghanaian Children

**DOI:** 10.3390/children13101313

**Published:** 2026-09-26

**Authors:** Niels Jansen, Emil van de Par, Prosper Moh, John Pettifor, Heleen Staal

**Affiliations:** 1 Department of Orthopaedic Surgery, MosaKids Children’s Hospital Maastricht UMC, P. Debeyelaan 25, P.O. Box 5800, 6202 AZ Maastricht, The Netherlands; niels_jig_jans@hotmail.com (N.J.); emilvdpar@gmail.com (E.v.d.P.); 2 Department of Orthopaedic Surgery, Saint John of God Hospital, Duayaw Nkwanta P.O. Box 24, Ghana; sjoghduayawnkwanta@chstgh.org; 3 Faculty of Health Sciences, University of the Witwatersrand, 7 York Road, Parktown, Johannesburg 2193, South Africa; john.pettifor@wits.ac.za

**Keywords:** rickets, varus, valgus, Africa, Blount

## Abstract

**Highlights:**

**What are the main findings?**

- In our Ghanaian patients, rickets was the predominant underlying cause of windswept deformity, although traumatic, dysplastic, and idiopathic causes were also identified.
- Windswept deformity usually developed shortly after the onset of walking and most commonly progressed from unilateral valgus to contralateral varus, supporting a multifactorial pathogenesis involving metabolic bone disease and mechanical loading.

**What are the implications of the main findings?**

- Children presenting with windswept deformity should undergo a structured diagnostic evaluation, including radiographic and biochemical assessment, to identify treatable underlying conditions such as rickets.
- Early recognition and management of metabolic bone disease may help prevent progression of deformity and improve long-term musculoskeletal outcomes, particularly in resource-limited settings.

**Abstract:**

Introduction: Windswept deformity is a malalignment of the knees characterised by unilateral valgus and contralateral varus. Only a limited number of studies have investigated the aetiology of windswept deformity. Most windswept cases in the literature are ancillary findings in studies or case reports. Typically, the causes of windswept deformity are classified into the following groups: ‘rickets and other metabolic disorders’, ‘skeletal dysplasia and other genetic causes’, ‘trauma’ and ‘idiopathic conditions’. Methods: After retrospectively reviewing patient files and radiographs, 23 patients with windswept deformity seen in a rural hospital in Ghana (between January 2017 and June 2022) were included. Data were collected through medical records, history taking, physical examination, and radiographs. Laboratory tests were carried out in 11 patients. Results: In most of the patients, windswept deformity developed shortly after the onset of walking (70% started within 6 months after walking); it started with unilateral valgus, which was later followed by contralateral varus (65%), and most patients had a normal weight (mean BMI 16.8, 51.7th percentile). A significant difference in mean BMI was observed when arm span was used instead of height (mean BMI: 15.8 ± 3.2 and 16.8 ± 4.0, respectively). The most common underlying condition was rickets (74%). Other causes include dysplastic (multiple epiphyseal dysplasia and unilateral Blount’s disease), post-traumatic (distal femur fracture), and idiopathic factors. Laboratory tests in 8 of the 11 patients showed vitamin D insufficiency. Conclusions: This study suggests that windswept deformity in young children usually develops with an initial valgus position, which is followed by contralateral varus. Rickets was the most frequently identified underlying diagnosis, with vitamin D insufficiency commonly observed among tested patients.

## 1. Introduction

Windswept deformity is a lower limb malalignment characterised by valgus deformity of one knee and varus deformity of the contralateral knee. In the literature, windswept deformity is most commonly described as a manifestation of an underlying condition rather than a disease entity in itself [1]. Most of the described patients in the literature with windswept deformity seem to have an African origin, particularly from the sub-Saharan regions [2,3,4]. The precise long-term consequences of the windswept deformity are not yet known. However, other similar untreated angular deformities of the knee, such as bowed and knocked knees, may lead to gait abnormalities, limb shortening and osteoarthritis [5].

The aetiology of windswept deformity is still not well understood. The deformity often arises shortly after a child starts walking. In 1980, Smyth indicated that the deformity starts with a rapid development of a valgus position of one knee due to an underlying pathology. The initial valgus position of the knee leads to a shift in weight to the medial compartment of the contralateral knee joint. Due to this shift and change in mechanical loading, a varus position on the contralateral side develops [2]. Most windswept cases in the literature are ancillary findings in studies or case reports. Since then, very few studies have been conducted on the aetiology of windswept deformity.

A recent systematic review classified the causes of windswept deformity in the following groups: ‘rickets and other metabolic disorders’, ‘skeletal dysplasia and other genetic causes’, ‘trauma’ and ‘idiopathic conditions’ [1]. Mechanical pressure, early walking, diet, and ethnicity were listed as possible factors contributing to the development of windswept deformity in the idiopathic group. The systematic review carried out by our research group has also presented a flow chart, designed to assist clinicians in finding the diagnosis of the possible underlying cause of windswept deformity.

The aim of this study was to describe the clinical presentation, progression, and underlying diagnoses of windswept deformity in children in Ghana and therefore create a better understanding of the aetiology of windswept deformity.

## 2. Materials and Methods

### 2.1. Study Design and Setting

This was a single-centre retrospective observational study with prospective cross-sectional reassessment of children with windswept deformity treated at St. John of God Hospital (Duayaw Nkwanta, Ghana) between January 2017 and June 2022. St. John of God Hospital is a private non-profit mission hospital, with 150 beds and specialising in orthopaedic surgery. This study adheres to the STROBE (Strengthening the Reporting of Observational Studies in Epidemiology) guidelines [6].

### 2.2. Participants

The orthopaedic theatre logbooks and the hospital’s records between January 2017 and June 2022 were searched for patients diagnosed with windswept deformity of the knees. Patients aged between 0 and 18 years were selected for the study. Both inpatients and outpatients were included. Patients were excluded when the diagnosis was not verified by the orthopaedic surgeon, the patient could not be contacted, or when no radiograph of the patient was available.

### 2.3. Data Collection and Statistical Analysis

Once informed consent was given, patients and their parents were invited to the hospital for an interview, physical examination, and additional investigations such as laboratory analyses. All radiographs were independently reviewed by the first three authors. In cases of disagreement, the findings were discussed until consensus was reached. Demographic information was collected from patient files and interviews. A standardised interview format was used to ask about general development, family history, past treatment or traumas, the origin of the windswept deformity, and any symptoms of chronic fluoride toxicity, hyperparathyroidism, skin disease or rickets. The same interpreter was used throughout all of the interviews.

Since the files only contained the weight at the time of diagnosis and not the height, the Body Mass Index (BMI) at the time of first presentation could not be obtained. During their subsequent visit, weight, length, and arm span were measured. Arm span was used as a substitute for measured height in BMI calculations because windswept deformity can cause a shortening in length of the patient’s legs, which could result in a falsely elevated BMI. All patients were between 2 and 19 years old (range: 2 and 10 years) at the time of anthropometric reassessment. The BMI Z-scores and percentiles using both height and arm span were determined using the ‘BMI-for-age for individuals aged 2–19 years’ scale issued by the U.S. Centers for Disease Control and Prevention (CDC) [7]. Underweight was defined as a percentile under the 5th (Z-score lower than −1.645), healthy weight as percentile between the 5th and 85th (Z-score −1.645 to 1.0), overweight as a percentile between 85th and 95th (Z-score 1.0 to 1.645), obese as a percentile above 95th (Z-score >1.645) and severely obese when BMI is >120% of the 95th percentile or ≥35 kg/m^2^.

Based on available radiographic data, it was documented which side had the varus and which side had the valgus alignment. Full-length lower limb or AP and lateral knee, weight-bearing radiographs were available for all included patients. During radiographic acquisition, patients were positioned with the patellae facing forward and the feet parallel to each other, as per hospital protocol. The anatomical tibiofemoral angle was used to quantify coronal lower-limb alignment using MicroDicom DICOM Viewer. The femoral anatomical axis was defined by a line through the centre of the femoral shaft, and the tibial anatomical axis by a line through the centre of the tibial shaft extending to the tibial eminence. The angle between these anatomical axes was recorded as the anatomical tibiofemoral angle. The average tibiofemoral angle is approximately 6° valgus [8]. Considering the normal physiological anatomical differences in children, a range of tibiofemoral angle between 0 and 10 degrees valgus was considered normal in this study [9]. The radiographic measurements were independently performed by the first two authors. The interobserver variation was minimal and therefore had no impact on outcomes.

Laboratory analysis was carried out by Lancet laboratories (https://www.lancet.co.za/ accessed on 1 March 2022). Analyses were performed on patients diagnosed less than one year prior to the first return visit (*n* = 10) or when no cause for windswept deformity was identified by the first authors (*n* = 1). The lab analysis included parathyroid hormone (PTH), biomarkers of rickets (25-hydroxyvitamin D, adjusted calcium, albumin, alkaline phosphatase and phosphate) and kidney function (creatinine). Lab tests for fluoride levels were not possible in the laboratories within the travel distance of the hospital. Continuous data are displayed as means ± standard deviations and categorical variables are shown as frequencies and/or as percentages. To compare BMI calculated using measured height and arm span, the distribution of the paired differences was assessed using the Shapiro–Wilk test. Because the paired differences were not normally distributed, the Wilcoxon signed-rank test was used to compare the two. Statistical significance was set at *p* < 0.05.

### 2.4. Diagnostic Classification

The underlying diagnosis of windswept deformity was classified based on the clinical history, physical examination, radiographic findings, and, when available, biochemical investigations.

Active rickets was diagnosed based on characteristic radiographic findings, including widening of the growth plate, metaphyseal cupping, fraying, and splaying. Patients with radiographically confirmed rickets who had previously been diagnosed and treated for rickets, but who showed residual metaphyseal growth disturbances consistent with a previous metabolic process, were classified as having healed rickets. Four patients with radiographically confirmed active rickets had no laboratory investigations because they had been diagnosed more than one year ago. Meanwhile, another four patients without laboratory testing were included in the healed-rickets group.

Biochemical findings were used to further characterise patients with radiographically confirmed rickets. Findings recorded included decreased 25-hydroxyvitamin D, decreased serum calcium, and decreased serum phosphate concentrations, together with alkaline phosphatase levels.

Blount’s disease was diagnosed radiographically according to the Langenskiöld criteria. Multiple epiphyseal dysplasia (MED) was classified as suspected MED when characteristic radiographic abnormalities, including irregular and delayed ossification at multiple epiphyses, were present; no genetic confirmation was available. Patients in whom no abnormality other than windswept deformity was identified on radiographic assessment were classified as having idiopathic deformity.

### 2.5. Ethical Approval

The Ghanaian medical ethics committee at Kwame Nkrumah University of Science and Technology (KNUST) gave ethical approval to perform this study (ref: CHRPE/AP/102/22). The study was carried out in accordance with the principles of the Declaration of Helsinki. Written informed consent was obtained from the patient, their parents, or legal guardian.

## 3. Results

A total of 36 patients with windswept deformity of the knee were selected, and after exclusion, a total of 23 patients were enrolled in the study. Two patients were excluded because the diagnosis was not confirmed by the orthopaedic surgeon; in eight cases, it was not possible to establish contact, and in three patients, no radiograph was present. Laboratory tests were obtained in eleven patients.

### 3.1. Patient Characteristics

At symptom onset, the mean age of the patients was 29 months ± 20 (12–96). Two-thirds of those patients were male. The majority of the patients were from the Akan tribe (*n* = 16, 70%). The remaining patients were from the Dagaaba, Ewe, Mole Dagbon, Kusasi, and Wangara tribes, and in one patient, the tribe was unknown. Most children with windswept deformity in our facility had undergone surgical correction (43%) or were waiting for surgery (22%). One-third had undergone conservative management. About half of our patients (48%) received supplementation using cod liver oil; all of those were treated before 2020, after which the treatment with cod liver oil was stopped. This was given when rickets was clinically suspected by the orthopaedic surgeon. The patients with supplementation were distributed across both conservative and surgical correction groups. Baseline characteristics are displayed in Table 1.

### 3.2. Interview and Physical Examination

The majority of patients (65%) reported that the deformity initially presented as a unilateral valgus deformity, which was followed by a varus deformity on the contralateral side. The mean age at walking onset was 17 months ± 5 mth (range: 9–24 mth). Sixteen patients (70%) developed windswept deformity within six months after the onset of walking. None of the patients reported a positive family history for windswept deformity. One patient reported a first-degree relative with bilateral valgus position of the knees, which was present since childhood. Two patients reported a second-degree relative with a unilateral valgus position, but this developed during adulthood. The medical history in one patient showed sickle cell disease, and one patient reported a fracture of the distal femur. The patient with the fracture of the distal femur developed a valgus position on the ipsilateral knee one month after treatment for the fracture using plaster of Paris. This valgus position was followed by a varus position on the contralateral side.

Although most patients had a normal BMI, there was a significant difference between the BMI measured using the patient’s height and arm span, with mean BMIs of 16.8 ± 4.0 and 15.8 ± 3.2, respectively. All 19 children had complete paired measurements. The paired differences were not normally distributed (Shapiro–Wilk W = 0.871, *p* = 0.015). Therefore, a Wilcoxon signed-rank test was used. The median paired difference between BMI calculated using arm span and BMI calculated using measured height was −0.8 (IQR −1.2 to −0.6). The Wilcoxon signed-rank test demonstrated a statistically significant difference between the two BMI estimates (W = 8, *p* < 0.001). On average, the arm span of patients was 3.3 cm [range −3, 8 cm] longer than their measured height. The BMI for age was normal in 14 out of 19 measured patients (74%); one patient (5%) was underweight, one was overweight (5%), two had obesity (11%), and one was severely obese (5%). When the BMI was calculated using the patient’s arm span, 13 patients out of the 19 measured patients (68%) had a normal BMI, three patients (16%) were underweight, one patient was overweight (5%), one had obesity (5%), and one was severely obese (5%). In five out of 19 patients (26%), evidence of active rickets was found during physical examination, and mild scoliosis was observed in one patient. One patient had poor dentition, which might be a symptom of chronic fluoride toxicity, but could very well be attributed to poor dental hygiene. Nonetheless, this patient had no other symptoms of fluoride toxicity besides a poor dental condition. Table 2 shows the results of the questionnaire and physical examination.

### 3.3. Additional Diagnostics

Radiographic images were examined for all 23 patients. The average angle of the valgus deformity on radiographs was 20.8° ± 7.0° (range: 12.5–35.5°), and for varus this was 14.1° ± 9.4° (range: 7.0–49.0°). The largest group of patients (*n* = 11, 48%) had active rickets as an underlying diagnosis. Six patients (26%) had evidence of a previous metaphyseal growth disturbance, most likely of metabolic origin (possible rickets), with a superimposed mechanical component. These findings represent a partially healed process with secondary mechanical loading. In combination with their history of rickets treatment two to four years previous, these six were diagnosed as healed rickets. Another patient was suspected to have MED based on the radiograph. Two patients had evidence of Blount’s disease on the radiographs. No abnormalities (other than windswept deformity) were detected on the radiographs of two patients at the time of diagnosis and were therefore considered idiopathic.

Laboratory tests were carried out in 11 patients who had been diagnosed within one year prior to inclusion or who did not have a clear diagnosis of the underlying pathology causing the windswept deformity. Blood tests performed on the nine patients with rickets showed lowered 25-hydroxyvitamin D levels in eight (89%) of them. One of these patients had both low vitamin D and calcium levels, and another patient had both vitamin D insufficiency and hypophosphatemia. In six of the nine rickets patients with blood tests (67%), the decreased vitamin D was associated with an increased value of PTH. Most patients with vitamin D insufficiency had an elevated alkaline phosphatase (86%). The patients suspected to have MED and the patient with Blount’s disease showed normal laboratory results. A lowered creatinine value was found in three of the eleven tested patients (27%).

Table 3 gives an overview of the underlying diagnoses for patients with windswept deformity. The laboratory and radiographic findings are presented in Table 4.

## 4. Discussion

This study provides additional insight into the clinical presentation, progression, and underlying diagnoses of windswept deformity in Ghanaian children. Rickets was identified as the predominant underlying condition, while most deformities developed shortly after the onset of walking and frequently started with unilateral valgus deformity before progression to a contralateral varus alignment.

The high prevalence of radiologically confirmed rickets with low 25-hydroxyvitamin D among the included patients suggests that metabolic bone disease plays an important role in the development of windswept deformity in this population. These findings are consistent with previous reports describing rickets as one of the leading causes of windswept deformity in sub-Saharan Africa [13,14,15]. Elevated alkaline phosphatase and secondary hyperparathyroidism in several patients further support the role of disturbed bone metabolism. Possible contributing factors include the increased sun exposure required for adequate vitamin D synthesis in children with darker skin pigmentation and cultural or religious skin covering. Previous studies have suggested that vitamin D deficiency is more common in younger children, whereas calcium deficiency-related rickets predominates at older ages [16,17]. This is consistent with our study population, in which all children with rickets with low 25-hydroxyvitamin D developed windswept deformity before four years of age, while patients with MED, Blount’s disease, rickets with hypophosphatemia, or idiopathic deformity presented at older ages ranging from 48 to 96 months.

The reported clinical history was consistent with the mechanical theory proposed by Smyth [2]. In 15 patients (65%), parents or patients reported that the deformity initially presented as unilateral valgus, followed by contralateral varus. Although this sequence was reported by parents or patients and not confirmed by serial radiographs, it is consistent with the proposed mechanism in which altered weight distribution across the contralateral limb may contribute to the development of varus deformity. Also, the observation that most children developed windswept deformity within six months after the onset of walking (70%) may further support the contribution of mechanical loading during early skeletal development [2,4,18].

Taken together, these findings support a multifactorial model in which metabolic bone disease and mechanical loading interact during a critical period of skeletal development. Rickets may weaken the growing physis and metaphysis, increasing susceptibility to deformity under physiological loading conditions. The reported sequence that the deformity frequently developed shortly after the onset of walking and often progressed from unilateral valgus to contralateral varus is consistent with the proposed mechanical hypothesis. Rather than representing competing explanations, metabolic and mechanical factors may act synergistically in the development of windswept deformity.

The identification of traumatic, dysplastic, and idiopathic causes highlights that windswept deformity should be regarded as a clinical phenotype rather than a diagnosis in itself. Consequently, clinicians should maintain a broad differential diagnosis and avoid assuming that all cases are attributable to rickets, even in regions where metabolic bone disease is common. Similarly, the identification of trauma [19], Blount’s disease [3,20,21] and MED [22] in our study population is consistent with previous reports describing these conditions as causes of windswept deformity.

Our findings provide additional clinical support for the diagnostic framework proposed in our previous systematic review [1]. Most underlying diagnoses could be identified through a structured assessment consisting of detailed history taking, physical examination, radiographic evaluation, and targeted biochemical investigations. In particular, our results highlight the importance of combining radiographic findings with biochemical evaluation. This is also in line with the current recommendations that the diagnosis of rickets should integrate clinical, biochemical, and radiographic assessment [23]. In several children, biochemical abnormalities supported the radiographic diagnosis despite limited clinical evidence of active rickets. This finding highlights the risk of underdiagnosing metabolic bone disease when relying solely on clinical examination. Although advanced genetic testing, fluoride measurements, and extensive metabolic investigations were not available, the majority of underlying diagnoses could still be identified using relatively accessible diagnostic tools. These findings suggest that a structured diagnostic approach may be clinically valuable, even in resource-constrained healthcare settings.

An interesting finding in this study was the significant difference between BMI calculated using measured height and BMI calculated using arm span. In our study population, the mean difference between height and arm span was 3.3 cm, which resulted in a significant difference in mean BMI. Previous studies have shown that arm span is a more reliable surrogate for height in patients with conditions associated with height loss, such as spinal deformities [24,25]. This comparison was considered exploratory, as arm span has not been validated as a substitute for height for BMI calculation in children with windswept deformity. Nonetheless, the observed difference between height-based and arm span-based BMI calculations may have implications for nutritional assessment in children with windswept deformity. To our knowledge, this issue has not previously been investigated in this patient population and therefore needs further investigation and validation.

This study has several strengths. It is one of the relatively few clinical studies specifically evaluating the aetiology of windswept deformity in sub-Saharan Africa and represents one of the larger contemporary cohorts of children with windswept deformity reported in the literature. Much of the existing literature focuses on a single underlying condition, such as rickets or achondroplasia, or is based on studies published several decades ago. In addition, few previous studies have systematically combined clinical history, physical examination, radiographic assessment, and biochemical investigations when evaluating children with windswept deformity. The inclusion of biochemical testing represents a particular strength of this study, as it provided additional evidence supporting the diagnosis of metabolic bone disease in several patients. The present study therefore contributes valuable clinical, radiographic, and biochemical data to the limited literature on windswept deformity and provides a more comprehensive evaluation of its underlying diagnosis in children.

Research in low-resource settings remains challenging due to limited facilities and data preservation. Several limitations should be acknowledged. First, due to the absence of an electronic medical record system, there was missing data. This resulted in more patients having to be excluded, with some data relying on self-reported retrospective information and missing anthropometric data in four cases. Therefore, there is a risk of recall bias. Secondly, the timing of biochemical assessment is a limitation. Laboratory investigations were performed during subsequent reassessment or at the time of diagnosis but not always at the time of deformity onset due to late presentation of some patients. Furthermore, some had received treatment for rickets several years before reassessment. Consequently, the biochemical findings, including 25-hydroxyvitamin D concentrations, may not accurately reflect the metabolic status at the time of deformity onset but rather the current metabolic status. Thirdly, cod liver oil was used as a supplement for rickets patients until 2020, which is inferior to Vitamin D supplementation. At that time, cod liver oil was the available and affordable drug in this rural area in Ghana, where vitamin D was not regularly available and/or was more expensive. Fourth, although the sample size was relatively small, windswept deformity is an uncommon condition, and this study population represents one of the larger contemporary studies specifically evaluating its aetiology. Nevertheless, the limited sample size reduces statistical power and may affect the generalizability of the findings. Furthermore, advanced genetic testing and fluoride measurements were unavailable, limiting evaluation of less common underlying diagnoses. Finally, this was a single-centre study, which may not fully represent the broader Ghanaian population.

Despite increasing recognition of windswept deformity, its pathogenesis remains incompletely understood. Future multi-centre prospective studies with larger patient populations and genetic analysis should be done to better understand the interaction between nutritional, mechanical, environmental, and genetic factors contributing to windswept deformity. Early recognition and treatment of rickets may play an important role in preventing progression of deformity and reducing long-term disability. The observed discrepancy between height-based and arm span-based BMI calculations suggests that nutritional assessment in children with lower limb deformities (e.g., Windswept deformity and Blount’s disease) may require alternative anthropometric measures. Future research should assess whether BMI calculated using height, arm span, or tibial length provides the most reliable estimation of nutritional status in this group of patients.

## 5. Conclusions

Windswept deformity in our Ghanaian study population was most commonly associated with rickets and frequently developed shortly after the onset of walking. The findings support a multifactorial aetiology and suggest a possible interaction in which metabolic bone disease increases susceptibility to deformity, while mechanical loading during early ambulation may contribute to its progression. Early recognition of underlying metabolic abnormalities may provide opportunities for prevention and timely treatment. These hypotheses require confirmation in prospective longitudinal studies with systematic biochemical, dietary, clinical, and serial radiographic assessment.

## Figures and Tables

**Table 1:** Baseline characteristics of patients included with windswept deformity (*n* = 23).

Gender	Male	15 (65%)
	Female	8 (35%)
Ethnic group	Akan	16 (70%)
	Dagaaba	2 (9%)
	Ewe	1 (4%)
	Mole Dagbon	1 (4%)
	Wangara	1 (4%)
	Kusasi	1 (4%)
	Missing information	1 (4%)
Age of onset in months	Mean	29 mth ± 20 mth (Range: 12–96).
	0–12 mth	2 (9%)
	13–24 mth	15 (65%)
	25–36 mth	3 (13%)
	37–48 mth	0 (0%)
	>49 mth	3 (13%)
Received treatment	Surgical correction	10 (43%)
	Waiting for surgery	5 (22%)
	Conservative treatment	8 (35%)
Supplementation	Cod liver oil	11 (48%)

**Table 2:** Questionnaire results (*n* = 23) and physical examination results (* *n* = 19) of patients included with windswept deformity (*n* = 23).

Medical history	None	21 (92%)
	Sickle cell disease	1 (4%)
	Fractured distal femur	1 (4%)
Initial presentation of windswept deformity	Valgus	15 (65%)
	Varus	3 (13%)
	Both	5 (22%)
Age at walking onset (months)	Mean	17 ± 5 (9–24)
	0–12	7 (30%)
	13–18	11 (48%)
	19–24	5 (22%)
Deformity developed after onset of walking (months)	0–3	11 (48%)
	4–6	5 (22%)
	7–12	0 (0%)
	13–24	3 (13%)
	>24	4 (17%)
Chronic Fluoride toxicity	None	23 (100%)
History of trauma	None	22 (96%)
	Fractured distal femur	1 (4%)
Signs of rickets *	Rickets rosary	4 (21%)
	Thickened wrists	1 (5%)
Skin disease *		0 (0%)
Skeletal deformity *	Scoliosis	1 (5%)
Primary hyperparathyroidism *		0 (0%)
BMI for age (using height) *	Mean BMI	16.8 ± 4.0 [12.5–31.7]
	Mean Z-score	0.2 ± 1.7 [−3.46–5.03]
	Mean percentile	51.7 ± 32.8 [0–100]
	Underweight	1 (5%)
	Normal BMI	14 (74%)
	Overweight	1 (5%)
	Obesity	2 (11%)
	Severely obesity	1 (5%)
BMI for age (using arm span as height) *	Mean BMI	15.8 ± 3.2 [12.2–27.3]
	Mean Z-score	−0.5 ± 1.9 [−3.99–4.49]
	Mean percentile	36.5 ± 33.3 [0–100]
	Underweight	3 (16%)
	Normal BMI	13 (68%)
	Overweight	1 (5%)
	Obesity	1 (5%)
	Severely obesity	1 (5%)

**Table 3:** Underlying diagnoses in patients with windswept deformity.

Underlying Diagnosis of Windswept Deformity	Subgroup	*n* = 23
Rickets	Total	17 (74%)
	Low 25-hydroxyvitamin D	5 (22%)
	Low 25-hydroxyvitamin D and low serum calcium	1 (4%)
	Hypophosphatemia and low 25-hydroxyvitamin D	1 (4%)
	Healed rickets (based on lab and radiograph)	6 (26%)
	Without biochemical assessment	4 (17%)
Trauma	Fractured distal femur	1 (4%)
Dysplasias or growth disorders	Signs of MED	1 (4%)
	Blount’s disease	2 (9%)
Unspecified/Idiopathic	No abnormalities on radiograph	2 (9%)

**Table 4:** Results of lab analysis combined with the radiograph results of included patients having windswept deformity.

Patient	Radiograph	Vitamin D (ng/mL) [10] <10: Deficiency 10–20: Insufficiency >20: Normal	PTH (pg/mL) [11] 14–35	Creatinine (umol/L) [12] 1–2 yrs: 15–31 3–4 yrs: 23–37 5–6 yrs: 25–42 7–8 yrs: 30–48 9–11 yrs: 29–56	Calcium Adjusted (mmol/L) [12] 2.20–2.70	Alkaline Phosphatase (IU/L) [12] 1–9 yr: 156–369 10–12 yr: 141–460	Phosphate (mmol/L) [12] 1–15 yr: 0.9–1.8
1	Rickets with hypophosphatemia	19 (-)	32	20 (-)	2.54	630 (+)	0.84 (-)
2	Healed rickets	27	24	21 (-)	2.50	231	1.50
3	Rickets with low 25-hydroxyvitamin D	17 (-)	85 (+)	21	2.41	365	2.14 (+)
4	Rickets with low 25-hydroxyvitamin D	15 (-)	93 (+)	17	2.41	539 (+)	1.47
5	Healed rickets	19 (-)	45 (+)	25	2.37	342	1.90 (+)
6	Rickets with low 25-hydroxyvitamin D	14 (-)	71 (+)	25	2.31	879 (+)	1.72
7	Rickets with low 25-hydroxyvitamin D	16 (-)	83 (+)	24	2.48	441 (+)	1.30
8	Rickets with low 25-hydroxyvitamin D	19 (-)	72 (+)	21	2.35	444 (+)	2.20 (+)
9	Rickets with low Vitamin D and low calcium	16 (-)	68 (+)	18 (-)	2.12 (-)	653 (+)	1.38
10	MED	23	27	28	2.56	218	1.50
11	Blount’s disease	32	21	43	2.36	357	1.08

## Data Availability

The data presented in this study are available on request from the corresponding author due to privacy concerns.
